# Genome-wide microRNA changes in human intracranial aneurysms

**DOI:** 10.1186/s12883-014-0188-x

**Published:** 2014-10-10

**Authors:** Dehua Liu, Liang Han, Xiao Wu, Xinjian Yang, Qunye Zhang, Fan Jiang

**Affiliations:** Department of Neurosurgery, China-Japan Union Hospital, Jilin University, Changchun, Jilin Province China; Key Laboratory of Cardiovascular Remodeling and Function Research, Qilu Hospital, 107 Wenhuaxi Road, Jinan, 250012 Shandong Province China; Beijing Neurosurgical Institute, Beijing Tiantan Hospital, Capital Medical University, Beijing, China

**Keywords:** Intracranial aneurysm, microRNA, Microarray, Human, Cerebral vascular disease, System biology, Transcriptome, Protein translation machinery

## Abstract

**Background:**

Intracranial aneurysms are pathological dilatations of the cerebral artery, while rupture of intracranial aneurysms causes life-threatening subarachnoid hemorrhage. The molecular mechanisms of pathogenesis of intracranial aneurysms are poorly understood. MicroRNAs have fundamental roles in modulating vascular biology and disease. In the present study, we carried out a genome-wide characterization on expressions of microRNAs, and performed integrative analyses in conjunction with changes of the transcriptome in human intracranial aneurysms.

**Methods:**

Genome-wide microRNA screening was performed in 6 intracranial aneurysmal samples and 6 normal superficial temporal arteries. Each case and control pair was individually matched with gender, age (±5 years), and high blood pressure history. Microarray analysis was performed using Agilent Human miRNA arrays.

**Results:**

As compared to normal arteries, we identified 157 microRNAs that were differentially expressed in the aneurysmal tissue (*P* < 0.05 and fold change ≥ 2), including 72 upregulated and 85 downregulated. The changed microRNAs included endothelium-enriched microRNAs such as members of the let-7 family, miR-17, miR-23b, miR-126, hsa-miR-24-1 and miR-222, and vascular smooth muscle-enriched miRNAs such as miR-143 and miR-145. Moreover, miR-1, miR-10a, miR-125b, and miR-26a, which were implicated in modulating vascular smooth muscle cell functions such as proliferation, apoptosis and shift of phenotype, were also changed. In contrast, microRNAs involved in monocyte and macrophage functions, such as miR-155, miR-146a, miR-223, and miR-124a, were not significantly changed. Bioinformatic analysis revealed that the changed microRNAs were associated with several biological processes related to aneurysm formation, including inflammation, dysregulation of extracellular matrix, smooth muscle cell proliferation, programmed cell death, and response to oxidative stress. Interestingly, we found that a subset of the potential microRNA target genes belonged to the protein translation machinery, including various eukaryotic translation initiation factors and ribosomal proteins, and this finding was highly correlated with our previous transcriptome data showing that multiple genes of the ribosomal proteins and translation initiation and elongation factors were significantly downregulated in human intracranial aneurysms.

**Conclusions:**

Our results support that dysregulated microRNAs may have a pathogenic role in intracranial aneurysms. Disruption of the protein translation process may have a pathogenic role in the development of intracranial aneurysms.

**Electronic supplementary material:**

The online version of this article (doi:10.1186/s12883-014-0188-x) contains supplementary material, which is available to authorized users.

## Background

Intracranial aneurysms (IAs) are pathological dilatations of the cerebral artery; rupture of IAs is the primary cause of life-threatening subarachnoid hemorrhage (SAH) [1-3]. The cellular and molecular mechanisms underlying the pathogenesis of IA formation are still poorly understood. Factors including smoking, hypertension, excessive alcohol consumption, vascular inflammation, nutritional factors and mechanical forces produced by the blood flow may all contribute to the formation and/or rupture of IAs [4-10]. On the other hand, mounting evidence has suggested that genetic factors (such as gene polymorphisms) also have important roles in the etiology of IAs [11,12].

Throughout IA research, identification of aberrantly expressed genes in IA tissues remains to be a core approach to understanding the molecular regulatory mechanisms underlying IA development and rupture. In line with this, several groups have employed high-throughput microarray methods to study gene expression changes at the whole genome level [13-17]. For example, in a previous study, we identified 1,160 genes whose expression levels were significantly changed in un-ruptured aneurysmal tissues as compared to normal blood vessels [15]. We found that a cluster of extracellular matrix related genes (including collagens type I, III, V, and XI and metalloproteinases) were significantly changed. Moreover, we found that a number of immune/inflammation-related genes were also differentially expressed in IA tissues [15]. Collectively, these functional genomic studies provided important information regarding the potential molecular mechanisms implicated in this multifactorial cerebral vascular disease [18].

MicroRNAs (miRNAs) are a class of short (18–25 nucleotides), non-coding RNAs that have fundamental roles in post-transcriptional regulation of gene expression. Regulation of gene expression by miRNAs may be achieved via either sequence-specific interactions with target mRNAs and subsequent mRNA degradation, or miRNA-mediated translational repression [19,20]. Therefore, miRNAs represent another layer of regulation of gene expression in addition to the conventional promoter-dependent transcriptional regulation. There is evidence showing that miRNAs are able to regulate a variety of target genes which are critical for the homeostasis of vascular endothelial and smooth muscle cells [21]. Consistent with this notion, experimental studies have revealed that aberrant expression of several miRNAs may be involved in pathological vascular remodeling, in which each individual miRNA may have a specific protective or pathogenic role [22,23]. In particular, different groups have shown that the expression levels of many miRNAs are significantly changed in human abdominal aortic aneurysm (AAA) tissues [24,25].

Currently, however, there is limited information about alterations in miRNA expression in cerebral aneurysms. A recent genomic study demonstrated that in human IA tissues, 18 miRNAs were significantly downregulated [26]. Despite these pilot results, however, there is evidence suggesting that the degree of correlation between different microarray data sets is low [18]. Hence, given the small sample number included in the previous study, a necessity for separate verification experiments is highlighted. In our previous study, we demonstrated that a subset of inflammation-related miRNAs were selectively upregulated in the plasma of stroke patients with intracerebral hemorrhage [27]. In the present study, we carried out experiments to detect genome-wide changes in miRNA expression in IAs, and performed integrative analyses in conjunction with changes of the IA transcriptome, in an attempt to identify novel biological processes that might be implicated in IA pathogenesis.

## Methods

### Patient recruitment

**Figure 1:**
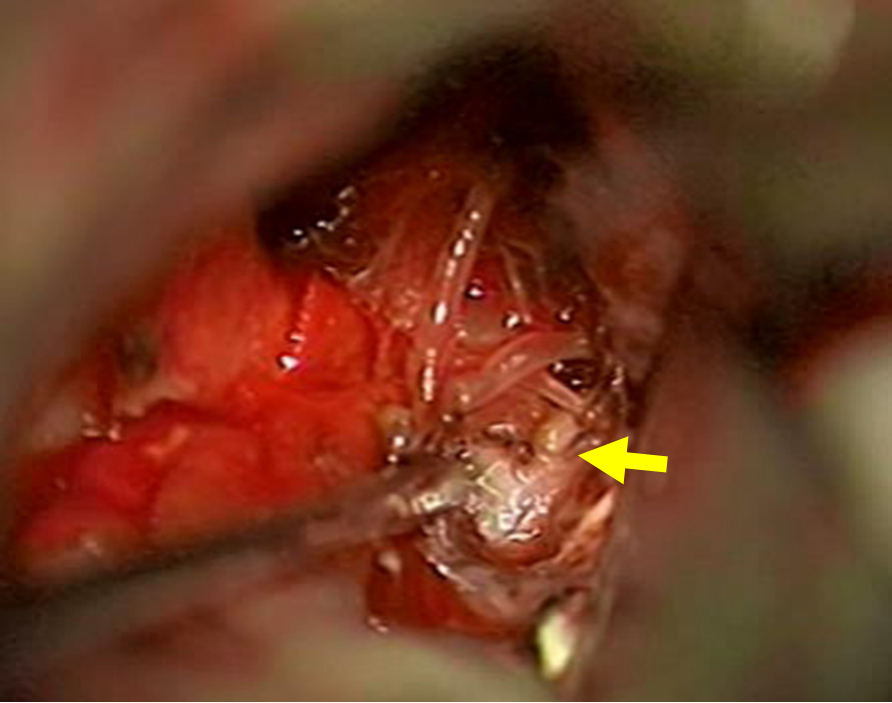
**A photo taken during surgery showing the gross pathology of an aneurysm (arrow) of the posterior communication artery.**

This study was approved (#10051) by the Human Ethics Committee of Shandong University Qilu Hospital, and informed consents were obtained before start of the experiment, directly from the patients or their first-degree relatives of unconscious subjects. Six patients with IAs undergoing surgical clipping treatment were included in the study. These cases were selected by the operating neurosurgeons on a basis that removal of the residual aneurysmal wall would not affect the outcome of the treatment. IA tissues were removed during clipping surgery (see Figure 1) and snap-frozen in liquid nitrogen. The basic clinical data of patients and characteristics of the aneurysms were summarized in Table 1. Normal superficial temporal arteries were collected from traumatic patients undergoing craniotomy treatments. Each case was individually matched in gender, age (±5 years), and high blood pressure history with controls. All of the control subjects were free of family history of aneurysmal disorders.

**Table 1:** **Clinical profiles of patients included in the microarray study and characteristics of the aneurysms**

**Sample #**	**Gender**	**Age range**	**Hypertension**	**Smoking history**	**Family history**	**Site of aneurysm**	**Type of aneurysm**	**Ruptured**
1*	F	60-65	Y	Y	N	Right internal carotid-posterior communicating artery	II	Y
2*	F	55-60	Y	N	N	Posterior communicating artery	II	Y
3	M	45-50	N	Y	N	Left middle cerebral artery-M2 segment	II	Y
4*	F	50-55	N	N	N	Left anterior cerebral artery	II	Y
5*	F	50-55	N	N	N	Left anterior cerebral artery-A2 segment	III	Y
6	F	45-50	N	N	N	Right posterior communicating artery	II	Y

*Samples #1 & #2 were combined before the microarray test because the yield of total RNA from each single sample was not enough for microarray detection. Samples #4 & #5 were also combined.

### Sample processing and RNA extraction

Frozen tissues were quickly transferred into TRIzol Reagent (Life Technologies, Grand Island, NY, USA) and homogenized immediately with a Dounce tissue grinder. Total RNA was isolated according to the manufacturer’s protocol and analyzed with Agilent Bioanalyzer 2100 (Agilent, Santa Clara, CA, USA). Because the total RNA yield from some aneurysmal tissues was below the minimum amount required for routine microarray detection, these low-yield samples were then combined to increase the mass of total RNA (see Table 1 footnote).

### Microarray assays

Microarray analysis was performed using Agilent Human miRNA (8*15K) V14.0 arrays (design ID: 31945) at ShanghaiBio Corporation (Shanghai, China). The miRNA molecules were labeled using the miRNA Complete Labeling and Hyb Kit from Agilent, following the manufacturer’s standard protocol. Each slide was hybridized with 100 ng Cy3-labeled RNA in hybridization oven at 55°C for 20 hours. After hybridization, slides were washed with Gene Expression Wash Buffer (Agilent) and scanned with an Agilent Microarray Scanner (G2565BA). Slide images were processed with the Feature Extraction software 10.7 (Agilent) with default settings.

### Bioinformatics analysis

Target genes for each miRNA were manually retrieved from different public databases, including miRecords [28], TarBase [29] and Ingenuity Knowledge Base (Ingenuity Systems, Redwood City, CA, USA). The miRNA target genes included in these databases are all experimentally validated and published in the literature. Gene functional annotations were performed using DAVID Bioinformatics Resources (National Institute of Allergy and Infectious Diseases, NIH) [30] or the IPA software (Ingenuity Systems). Benjamini and Hochberg False Discovery Rate analysis was used for multiple testing correction. The interaction networks were constructed using the Cytoscape platform based on information stored in public databases including BIND, DIP, HPRD, INTACT and BIOGRID [31]. The IA transcriptome data from our previous study were retrieved from GEO database (GSE26969) [15].

### Quantitative real-time PCR (qPCR)

Total RNA of 10 ng was converted to cDNA using miRCURY LNA Universal cDNA Synthesis Kit (Exiqon). The cDNA was diluted 20 times and 4 μl of diluted cDNA was added into a 10 μl reaction system for qPCR reaction. qPCR was performed with the pre-designed UniRT LNA microRNA primer sets and SYBR Green master mix (all from Exiqon). The catalogue numbers of the primers for miR-99b*, miR-340*, miR-493 and miR-1208 were 204064, 204250, 204557 and 204537 respectively. The sequence for miR-648 was aagugugcagggcacuggu. U6 and RNU5G snRNAs (Exiqon Cat #203907 and 203908) were used as house-keeping genes. To ensure that the UniRT LNA primer-based PCR reactions had consistent amplification efficiency, we performed characterization experiments by amplifying serial dilutions of synthetic miRNA templates. We confirmed that there was a nice linearity between the log-transformed concentration of input miRNA and the yielding Ct values (r^2^ > 0.96). The relative quantification method was used for qPCR data analysis [32]. Principally, ΔCt was calculated as the Ct value of the test gene minus that of the house-keeping gene. ΔΔCt was calculated as the ΔCt value of a given gene in patient subjects minus that in control subjects. The relative gene expression level (fold) was expressed as the calculated value of 2^-ΔΔCt^.

### Statistical analysis

The raw microarray data were processed and analyzed with the GeneSpring GX software (Agilent). The data were first filtered according to the signal intensities, with genes that were classified as A (absence) in all samples being excluded from further analysis. The Robust Multi-Array Average (RMA) algorithm was used for data normalization, and the Benjamini and Hochberg False Discovery Rate multiple testing correction was used for statistical analysis. Other data were expressed as mean ± standard error of the mean (SEM). Differences between groups were analyzed with unpaired *t*-test using GraphPad Prism. A value of *P* < 0.05 was regarded as statistically significant.

## Results

**Figure 2:**
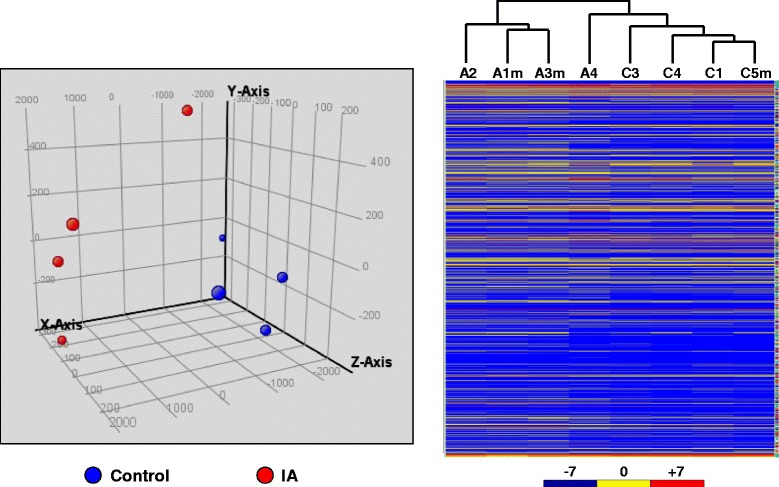
**Principal component analysis (left) and hierarchical clustering analysis (right) of the microarray data.** In the principal component graph, each dot represents a single array. In the hierarchical clustering graph, C represents control tissues and A represents aneurysmal tissues.

We profiled global miRNA expression levels in IA tissues and compared with normal blood vessels. Out of the 887 miRNA genes included in the array, 475 miRNAs were detected (not classified as Absence in at least one of the samples tested). Principal component analysis (PCA) of the microarray data showed that IA samples and control samples displayed divergent trends in signal intensity distributions as shown in Figure 2, indicating an altered miRNA expression profile in IAs. Likewise, hierarchical clustering analysis clearly demonstrated that the patterns of global miRNA expression were distinct between IAs and control vessels (with all IA samples and control samples being clustered together respectively) (Figure 2). Specifically, we identified 157 miRNAs that were differentially expressed in IAs (*P* < 0.05 and fold change ≥ 2), including 72 upregulated and 85 downregulated versus the control vessel (see Addition files 1 & 2).

Among the changed miRNAs, we observed a number of miRNAs that were primarily involved in functions of vascular endothelial and smooth muscle cells. These included endothelium-enriched miRNAs such as members of the let-7 family (let-7a to let-7g, and let-7i), miR-17, miR-23b, miR-126, hsa-miR-24-1 and miR-222 [25,33]; and vascular smooth muscle-enriched miRNAs such as miR-143 and miR-145 [34,35]. Moreover, the changed miRNAs also contained miR-1, miR-10a, miR-125b, and miR-26a, which were implicated in modulating vascular smooth muscle cell functions such as proliferation, apoptosis and shift of phenotype [34,35]. In contrast, miRNAs involved in monocyte and macrophage functions, such as miR-155, miR-146a, miR-223, and miR-124a, were not significantly changed [25].

**Figure 3:**
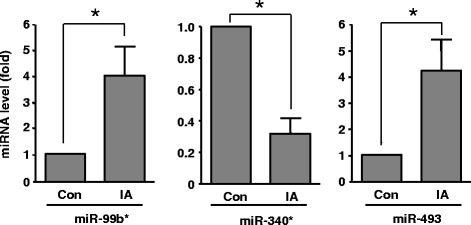
**Relative levels of three randomly selected miRNAs in control and aneurysmal tissues as measured by quantitative real-time PCR.** *P < 0.05, unpaired *t*-test, *n* = 7 - 9. Data are expressed as mean ± SEM.

To validate the microarray data, we randomly selected five miRNAs, including miR-99b*, miR-340*, miR-493, miR-1208 and miR-648, for qPCR validation (all *P* values for these miRNAs in the microarray assay being < 0.001). In our preliminary test, however, we found that miR-648 and miR-1208 could not be readily detected by PCR, hence we did not include them in further analysis. For PCR experiments, we used a semi-independent sample including new and the original samples, because of the shortage of clinical IA specimens. Basic clinical parameters of this cohort were: average age 58 ± 3 years, 38% male, 25% with hypertension history, and 38% with smoking. We confirmed that miR-99b* and miR-493 were significantly upregulated in IAs, while miR-340* was downregulated (Figure 3). The trends of change in these miRNAs were consistent with those observed by microarray assays. To exclude the possibility that the selection of house-keeping gene could influence the qPCR results [36], we synthesized a *C. elegans* miRNA cel-miR-39-3p (by TaKaRa, Dalian, China) and used it as a spike-in reference. We determined that neither the U6 gene nor RNU5G gene was significantly different between control and IA samples (1.2 ± 0.5 and 1.3 ± 0.5 fold of controls for U6 and RNU5G respectively, all *P* > 0.05, *n* = 5). In addition, using Cq values of RNU5G as the house-keeping gene did not modify the trend of changes in gene expression as using U6.

To clarify whether and how these changed miRNAs were relevant to the development of IA, we performed bioinformatic data mining by surveying different public databases, and produced a target gene list for all of the differentially expressed miRNAs. Gene functional annotation analysis indicated that multiple biological processes/pathways associated to these miRNAs (and their target genes) were potentially connected to IA formation and/or rupture. These biological processes/pathways include blood vessel development, smooth muscle cell proliferation, programmed cell death, response to oxidative stress, extracellular matrix organization, transforming growth factor (TGF)-β signaling pathway, innate immune response, and leukocyte activation. The miRNA entities under each category and their target genes relevant to IA are summarized in Table 2. Of note, a predicted vascular abnormality that was associated with the changed miRNAs was aortic dissection, another aneurysmal disorder of the artery.

**Table 2:** **Functional classification of changed miRNAs in IA tissues and their target genes**

**Biological processes/disorders**	**Changed miRNAs**	**Corresponding target genes**
**Programmed cell death**	miR-29b, let-7a, miR-125a-5p, miR-199b-5p, miR-1, miR-30e*, miR-30c, miR-338-3p, miR-133a, miR-101, miR-26a, miR-362-3p, miR-362-5p, miR-330-3p, miR-296-5p, miR-139-5p, miR-103, miR-218	TNFRSF10B, TP53, BAK1, CASP6, CASP7, BCL2, CASP3, CASP9, MAP2K7, PTEN, BAX, AKT2, MAP2K4, MAPK1, MAPK3, PIK3R1, BNIP3L, BECN1
**Extracellular matrix organization**	miR-1, miR-30e*, miR-30c, miR-133a, let-7a, miR-199b-5p, miR-29b, miR-218	SERPINB5, CTGF, COL5A3, COL1A2, COL3A1, COL1A1, TGFBR1, TGFB3, SMAD3, MMP1, COL15A1, COL4A1, COL4A2, COL5A2, FBN1, SPARC
**Response to oxidative stress**	miR-133a, miR-30c, miR-199b-5p, miR-125a-5p, miR-1, let-7a, miR-101, miR-338-3p, miR-29b, miR-218, miR-26a, miR-296-5p, miR-139-5p	SIRT1, TXN2, HIF1A, GSS, SOD2, HMOX1, FOXO1,
**TGF-beta signaling pathway**	miR-125a-5p, miR-29b, miR-30c, miR-26a, miR-30e*, miR-1, let-7a, miR-218, miR-133a, miR-296-5p, miR-338-3p, miR-362-3p	ID2, ID1, ID3, ACVR2A, ACVR1, SMAD5, SMAD1, BMPR1B, BMPR2, TGFBR1, TGFBR2, SMAD4, TGFB3, SMAD3
**Smooth muscle cell proliferation**	miR-29b, miR-125a-5p, miR-1, let-7a, miR-101, miR-26a, miR-30c	KLF4, ID2, IGFBP3, PPARG, NOTCH3, IGF1, VEGFA, PTK2, JUN
**Aortic dissection**	miR-29b, miR-218, let-7a, miR-26a	COL3A1, COL1A1, TGFBR1, TGFBR2, SMAD3, COL5A2, FBN1

The target genes listed are all experimentally validated and published in the literature.

**Figure 4:**
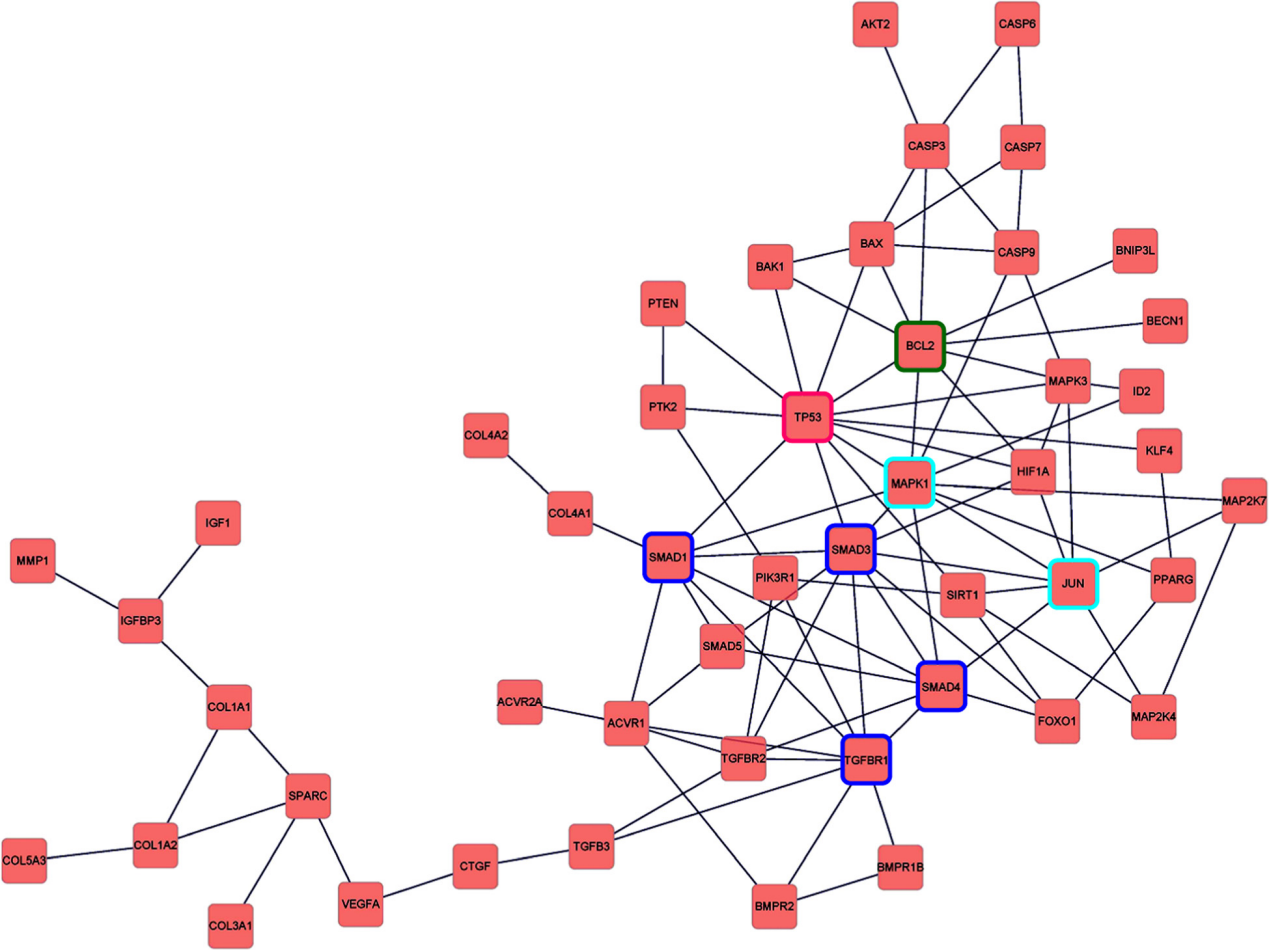
**Potential functional interactions of the target genes of the differentially expressed miRNAs.** Genes predicted to be with the most important functional roles (*i.e.* with the highest number of connections in the network) were highlighted in different colors.

To elucidate functional interrelationships of the miRNA target genes, we performed gene network analysis and created a network map covering a subset of the genes involved (Figure 4). We showed that there were complex functional interactions between the target genes. In particular, we identified several genes that might have the most important functional roles in the network (*i.e.* those with the highest number of connections with other genes), including p53, Bcl-2, Smad1/3/4, TGF-β receptor (TGFBR) 1, MAPK1 (mitogen-activated protein kinase 1, also known as ERK2) and c-Jun (Figure 4).

Bioinformatic analysis revealed that a subset of the potential miRNA target genes belonged to the protein translation machinery, including various eukaryotic translation initiation factors and ribosomal proteins (Table 3). Notably, this finding was highly correlated with our previous transcriptome study with a similar experimental design [15], showing that multiple genes of the ribosomal proteins and translation initiation and elongation factors were significantly downregulated in human intracranial aneurysms (see Table 3).

**Table 3:** **Genes related to eukaryotic protein translation identified by genomic miRNA and mRNA analyses**

**Potential target genes of the altered miRNAs in IA**	Downregulated mRNAs in IA*
Eukaryotic translation initiation factor 1 (EIF1)	Eukaryotic translation initiation factor 1A, X-linked (EIF1AX)
Eukaryotic translation initiation factor 1A, X-linked (EIF1AX)	Eukaryotic translation initiation factor 2, subunit 1 (EIF2S1)
Eukaryotic translation initiation factor 2, subunit 1 alpha (EIF2S1)	Eukaryotic translation initiation factor 3, subunit 4 (EIF3S4)
Eukaryotic translation initiation factor 2, subunit 2 beta (EIF2S2)	Eukaryotic translation initiation factor 3, subunit 7 (EIF3S7)
Eukaryotic translation initiation factor 3, subunit H (EIF3H)	Eukaryotic translation initiation factor 3, subunit 9 (EIF3S9)
Eukaryotic translation initiation factor 4A1 (EIF4A1)	Eukaryotic translation initiation factor 4B (EIF4B)
Eukaryotic translation initiation factor 4E binding protein 2 (EIF4EBP2)	Eukaryotic translation initiation factor 4E member 3 (EIF4E3)
Eukaryotic translation initiation factor 4 gamma, 3 (EIF4G3)	Eukaryotic translation elongation factor 1 delta (EEF1D)
Ribosomal protein L32 (RPL32)	Ribosomal protein L10 (RPL10)
Ribosomal protein L9 (RPL9)	Ribosomal protein L18 (RPL18)
Ribosomal protein S23 (RPS23)	Ribosomal protein L19 (RPL19)
Ribosomal protein S4 (RPS4Y1)	Ribosomal protein L3 (RPL3)
Ribosomal protein S6 kinase, 90kDa, polypeptide 1 (RPS6KA1)	Ribosomal protein L35a (RPL35A)
	Ribosomal protein L36 (RPL36)
	Ribosomal protein L8 (RPL8)
	Ribosomal protein S14 (RPS14)
	Ribosomal protein S15 (RPS15)
	Ribosomal protein S3 (RPS3)
	Ribosomal protein S7 (RPS7)
	Ribosomal protein S6 kinase, 90kDa, polypeptide 5 (RPS6KA5)

*The mRNA data were obtained by reanalysis of our previous data set (GEO accession #GSE26969).

## Discussion

In this study, we compared miRNA expression profiles in human IAs and normal arterial tissues. We have discovered that there are extensive changes in miRNA expression in IAs, while the biological functions of the majority of these miRNAs remain to be clarified. In a recent study in ruptured IA samples, the authors reported that 18 miRNAs were significantly downregulated in IAs; these miRNAs were associated with functions including proliferation and migration of leukocytes and/or smooth muscle cells [26]. In the present study, we identified 85 downregulated miRNAs in the IA tissue. Notably, ~ 50% of the downregulated miRNAs reported by the previous study have also been confirmed by our results. However, we also identified 72 miRNAs that were upregulated in IAs, while no miRNA upregulation was detected in the previous study [26]. As suggested, the difference in the number of altered miRNAs detected in the two studies is likely to be due to the small sample size used for microarray analysis [18]. It was noted that only 3 IA specimens were used in the initial microarray discovery stage in the previous study, and this limited biological replication might compromise the rate of positive findings [26].

Genomic annotation analysis has provided some clues on the potential functional connections between the altered miRNAs and IA pathogenesis. Specifically, network analysis revealed that the altered miRNAs were related to several biological processes, including innate immune response, leukocyte activation, extracellular matrix organization, TGF-β signaling, smooth muscle cell proliferation, blood vessel development, programmed cell death, and response to oxidative stress. Of note, these biological functions are closely associated with multiple pathological conditions such as inflammation, dysregulation of extracellular matrix, and disrupted blood vessel homeostasis, all of which are closely associated with the development of IA [7,37-40]. Moreover, a subset of the changed miRNAs (e.g. miR-1, miR-125b, miR-222, miR-26a, miR-17, miR-126 and miR-23b) have been shown to have important roles in modulating functions of vascular endothelial and smooth muscle cells, and dysregulation of these miRNAs may be associated with various vascular pathologies [33-35]. Also, our results indicate that some of these miRNAs may be involved in modulating the turnover of extracellular matrix. For example, we showed that miR-29b was downregulated in IA. In a recent study, Fang et al. demonstrated that one of the direct gene targets of miR-29b was metalloproteinase-2 [41]. It is well documented that aberrant production or activation of metalloproteinases may have a critical role in IA development [42].

A causal role of altered miRNA expression in the pathogenesis of IA remains to be confirmed. In parallel, studies in animal models showed that interventions targeting specific miRNAs might have beneficial effects on the development of AAA [43]. Pahl et al. examined the miRNA expression pattern in human AAA tissues and revealed that miR-133a, miR-133b and miR-331-3p were significantly downregulated in AAAs [24]. Notably, we found that these three miRNAs were also downregulated in IAs, suggesting that they might be involved in some common pathways implicated in arterial aneurysm formation. Dysregulated expression of miR-133a and miR-133b has been found in some types of cancer [44]. Moreover, miR-133a has been shown to have critical roles in modulating cardiac development and maintaining skeletal muscle homeostasis [45,46]. Similar to miR-133, miR-331-3p is also involved in regulating growth of certain cancer cells [47]. However, the biological effects of changed expressions of miR-133a/b and miR-331-3p in vascular tissues are currently unclear. In addition, we noted that the majority of changed miRNAs in human AAAs were distinct from those changed in IAs, supporting the argument that AAA and IA might be associated with divergent genetic and/or biochemical mechanisms [48].

An interesting finding was that there was a remarkable correlation between the present genomic miRNA data with our previous IA transcriptome data, showing that a subset of genes related to the protein translation process might be dysregulated in IAs. Indeed, abnormalities of the protein translation machinery underlie a variety of inheritable diseases [49]. Aberrant ribosomal biogenesis has also been linked to cardiovascular pathologies such as myocyte hypertrophy [50,51]. In vascular smooth muscle cells, activation of the Akt-mTOR (mammalian target of rapamycin)-p70S6 kinase pathway, the master regulator of eukaryotic protein translation, has profound impacts on the proliferation and differentiation functions [52-54]. Nevertheless, it remains to be clarified whether disruptions of the protein translational process are involved in the pathogenesis of IA.

Functional network analysis on the miRNA target genes suggests that p53, Bcl-2, and proteins involved in the TGF-β signaling pathway and the MAPK signaling pathway may be important regulators in the process of IA development. Both p53 and Bcl-2-related proteins have critical roles in modulating cell apoptosis and senescence [55,56]. Indeed, cell apoptosis appears to be a hallmark of the pathological changes occurring in the aneurysmal vessel wall [7,57]. Similarly, previous studies have shown that activation of the MAPK pathway, especially the JNK and p38 kinases, is also increased in IA tissues [58,59]. Given the fundamental roles of JNK and p38 in mediating cell apoptosis and inflammatory responses, and the close relationship between apoptosis/inflammation and IA [7], it is supposed that aberrant activation of the MAPK pathway may have an important role in the pathogenesis of IA [60]. For proteins involved in the TGF-β signaling pathway, limited evidence has suggested that the expression levels of TGF-β receptors may be changed during IA development [61]. It is known that genetic variations in the TGFBR1 and TGFBR2 genes are associated with an increased risk of AAA; however, there is no evidence that TGFBR1 or TGFBR2 gene polymorphisms are linked to IA [62]. Hence, an involvement of the TGF-β signaling pathway in IAs is still elusive.

A limitation of the present study is the relative small sample number, which is due to the difficulty in collecting suitable clinical IA specimens [63]. The aneurysmal samples included in the present study were obtained with cautions to guarantee that removal of the residual aneurysmal wall after clipping would have no impacts on the outcome of surgical treatment. Likewise, normal cerebral arterial tissues are not readily accessible for many researchers either. Hence, different groups used either superficial temporal arteries or middle meningeal arteries as control in array studies [13,15-17]. In the present study, we could not totally rule out differences in miRNA expressions between the superficial temporal artery and normal cerebral artery. Moreover, the microarray assay cannot provide information on the absolute copy number of a given miRNA, while this disadvantage can be overcome by more advanced technologies such as high-throughput sequencing.

## Conclusions

In this study, we showed that there were extensive changes in miRNA expression in human intracranial aneurysms, and these included several endothelium- and vascular smooth muscle-enriched miRNAs. Our results suggest that development of intracranial aneurysms may be associated with disruptions of the normal protein translational process in vascular cells.

### Availability of supporting data

More supporting data for microarray results are included as additional files (Additional file 1: Tables S1 and Additional file 2: Table S2).

## Additional files

Additional file 1: Table S1. MicroRNAs upregulated in human intracranial aneurysm. Additional file 2: Table S2. MicroRNAs downregulated in human intracranial aneurysm.

## Abbreviations

IA: Intracranial aneurysm
SAH: Subarachnoid hemorrhage
miRNA: microRNA
AAA: Abdominal aortic aneurysm
SEM: Standard error of the mean
PCA: Principal component analysis
MAPK: Mitogen-activated protein kinase
TGF: Transforming growth factor
TGFBR: TGF-β receptor

## References

[1] Krings T, Mandell DM, Kiehl TR, Geibprasert S, Tymianski M, Alvarez H, ter Brugge KG, Hans FJ (2011). Intracranial aneurysms: from vessel wall pathology to therapeutic approach. Nat Rev Neurol.

[2] Brown RD (2010). Unruptured intracranial aneurysms. Semin Neurol.

[3] Steiner T, Juvela S, Unterberg A, Jung C, Forsting M, Rinkel G (2013). European Stroke Organization guidelines for the management of intracranial aneurysms and subarachnoid haemorrhage. Cerebrovasc Dis.

[4] Wardlaw JM, White PM (2000). The detection and management of unruptured intracranial aneurysms. Brain.

[5] Clarke M (2008). Systematic review of reviews of risk factors for intracranial aneurysms. Neuroradiology.

[6] Juvela S, Poussa K, Porras M (2001). Factors affecting formation and growth of intracranial aneurysms: a long-term follow-up study. Stroke.

[7] Chalouhi N, Ali MS, Jabbour PM, Tjoumakaris SI, Gonzalez LF, Rosenwasser RH, Koch WJ, Dumont AS (2012). Biology of intracranial aneurysms: role of inflammation. J Cereb Blood Flow Metab.

[8] Sforza DM, Putman CM, Cebral JR (2009). Hemodynamics of Cerebral Aneurysms. Annu Rev Fluid Mech.

[9] Omodaka S, Sugiyama S, Inoue T, Funamoto K, Fujimura M, Shimizu H, Hayase T, Takahashi A, Tominaga T (2012). Local hemodynamics at the rupture point of cerebral aneurysms determined by computational fluid dynamics analysis. Cerebrovasc Dis.

[10] Shiue I, Arima H, Hankey GJ, Anderson CS (2011). Dietary intake of key nutrients and subarachnoid hemorrhage: a population-based case-control study in Australasia. Cerebrovasc Dis.

[11] Ruigrok YM, Rinkel GJ, Wijmenga C (2005). Genetics of intracranial aneurysms. Lancet Neurol.

[12] Caranci F, Briganti F, Cirillo L, Leonardi M, Muto M (2013). Epidemiology and genetics of intracranial aneurysms. Eur J Radiol.

[13] Pera J, Korostynski M, Krzyszkowski T, Czopek J, Slowik A, Dziedzic T, Piechota M, Stachura K, Moskala M, Przewlocki R, Szczudlik A (2010). Gene expression profiles in human ruptured and unruptured intracranial aneurysms: what is the role of inflammation?. Stroke.

[14] Krischek B, Kasuya H, Tajima A, Akagawa H, Sasaki T, Yoneyama T, Ujiie H, Kubo O, Bonin M, Takakura K, Hori T, Inoue I (2008). Network-based gene expression analysis of intracranial aneurysm tissue reveals role of antigen presenting cells. Neuroscience.

[15] Li L, Yang X, Jiang F, Dusting GJ, Wu Z (2009). Transcriptome-wide characterization of gene expression associated with unruptured intracranial aneurysms. Eur Neurol.

[16] Shi C, Awad IA, Jafari N, Lin S, Du P, Hage ZA, Shenkar R, Getch CC, Bredel M, Batjer HH, Bendok BR (2009). Genomics of human intracranial aneurysm wall. Stroke.

[17] Marchese E, Vignati A, Albanese A, Nucci CG, Sabatino G, Tirpakova B, Lofrese G, Zelano G, Maira G (2010). Comparative evaluation of genome-wide gene expression profiles in ruptured and unruptured human intracranial aneurysms. J Biol Regul Homeost Agents.

[18] Roder C, Kasuya H, Harati A, Tatagiba M, Inoue I, Krischek B (2012). Meta-analysis of microarray gene expression studies on intracranial aneurysms. Neuroscience.

[19] Bartel DP (2004). MicroRNAs: genomics, biogenesis, mechanism, and function. Cell.

[20] He L, Hannon GJ (2004). MicroRNAs: small RNAs with a big role in gene regulation. Nat Rev Genet.

[21] Bonauer A, Boon RA, Dimmeler S (2010). Vascular microRNAs. Curr Drug Targets.

[22] McDonald RA, Hata A, MacLean MR, Morrell NW, Baker AH (2012). MicroRNA and vascular remodelling in acute vascular injury and pulmonary vascular remodelling. Cardiovasc Res.

[23] Wei Y, Schober A, Weber C (2013). Pathogenic arterial remodeling: the good and bad of microRNAs. Am J Physiol Heart Circ Physiol.

[24] Pahl MC, Derr K, Gäbel G, Hinterseher I, Elmore JR, Schworer CM, Peeler TC, Franklin DP, Gray JL, Carey DJ, Tromp G, Kuivaniemi H (2012). MicroRNA expression signature in human abdominal aortic aneurysms. BMC Med Genomics.

[25] Kin K, Miyagawa S, Fukushima S, Shirakawa Y, Torikai K, Shimamura K, Daimon T, Kawahara Y, Kuratani T, Sawa Y (2012). Tissue- and plasma-specific MicroRNA signatures for atherosclerotic abdominal aortic aneurysm. J Am Heart Assoc.

[26] Jiang Y, Zhang M, He H, Chen J, Zeng H, Li J, Duan R (2013). MicroRNA/mRNA profiling and regulatory network of intracranial aneurysm. BMC Med Genomics.

[27] Guo D, Liu J, Wang W, Hao F, Sun X, Wu X, Bu P, Zhang Y, Liu Y, Liu F, Zhang Q, Jiang F (2013). Alteration in Abundance and Compartmentalization of Inflammation-Related miRNAs in Plasma After Intracerebral Hemorrhage. Stroke.

[28] Xiao F, Zuo Z, Cai G, Kang S, Gao X, Li T (2009). miRecords: an integrated resource for microRNA-target interactions. Nucleic Acids Res.

[29] Vergoulis T, Vlachos IS, Alexiou P, Georgakilas G, Maragkakis M, Reczko M, Gerangelos S, Koziris N, Dalamagas T, Hatzigeorgiou AG (2012). TarBase 6.0: capturing the exponential growth of miRNA targets with experimental support. Nucleic Acids Res.

[30] da Huang W, Sherman BT, Lempicki RA (2009). Systematic and integrative analysis of large gene lists using DAVID bioinformatics resources. Nat Protoc.

[31] Kohl M, Wiese S, Warscheid B (2011). Cytoscape: software for visualization and analysis of biological networks. Methods Mol Biol.

[32] Livak KJ, Schmittgen TD (2001). Analysis of relative gene expression data using real-time quantitative PCR and the 2(-Delta Delta C(T)) Method. Methods.

[33] Santoro MM, Nicoli S (2013). miRNAs in endothelial cell signaling: the endomiRNAs. Exp Cell Res.

[34] Robinson HC, Baker AH (2012). How do microRNAs affect vascular smooth muscle cell biology?. Curr Opin Lipidol.

[35] Xie C, Zhang J, Chen YE (2011). MicroRNA and vascular smooth muscle cells. Vitam Horm.

[36] Vandesompele J, De Preter K, Pattyn F, Poppe B, Van Roy N, De Paepe A, Speleman F (2002). Accurate normalization of real-time quantitative RT-PCR data by geometric averaging of multiple internal control genes. Genome Biol.

[37] Penn DL, Witte SR, Komotar RJ, Sander Connolly E (2014). The role of vascular remodeling and inflammation in the pathogenesis of intracranial aneurysms. J Clin Neurosci.

[38] Bruno G, Todor R, Lewis I, Chyatte D (1998). Vascular extracellular matrix remodeling in cerebral aneurysms. J Neurosurg.

[39] Starke RM, Chalouhi N, Ali MS, Jabbour PM, Tjoumakaris SI, Gonzalez LF, Rosenwasser RH, Koch WJ, Dumont AS (2013). The role of oxidative stress in cerebral aneurysm formation and rupture. Curr Neurovasc Res.

[40] Frosen J, Tulamo R, Paetau A, Laaksamo E, Korja M, Laakso A, Niemela M, Hernesniemi J (2012). Saccular intracranial aneurysm: pathology and mechanisms. Acta Neuropathol.

[41] Fang JH, Zhou HC, Zeng C, Yang J, Liu Y, Huang X, Zhang JP, Guan XY, Zhuang SM (2011). MicroRNA-29b suppresses tumor angiogenesis, invasion, and metastasis by regulating matrix metalloproteinase 2 expression. Hepatology.

[42] Aoki T, Kataoka H, Morimoto M, Nozaki K, Hashimoto N (2007). Macrophage-derived matrix metalloproteinase-2 and -9 promote the progression of cerebral aneurysms in rats. Stroke.

[43] Maegdefessel L, Azuma J, Toh R, Merk DR, Deng A, Chin JT, Raaz U, Schoelmerich AM, Raiesdana A, Leeper NJ, McConnell MV, Dalman RL, Spin JM, Tsao PS (2012). Inhibition of microRNA-29b reduces murine abdominal aortic aneurysm development. J Clin Invest.

[44] Nohata N, Hanazawa T, Enokida H, Seki N (2012). microRNA-1/133a and microRNA-206/133b clusters: dysregulation and functional roles in human cancers. Oncotarget.

[45] Liu N, Bezprozvannaya S, Williams AH, Qi X, Richardson JA, Bassel-Duby R, Olson EN (2008). microRNA-133a regulates cardiomyocyte proliferation and suppresses smooth muscle gene expression in the heart. Genes Dev.

[46] Liu N, Bezprozvannaya S, Shelton JM, Frisard MI, Hulver MW, McMillan RP, Wu Y, Voelker KA, Grange RW, Richardson JA, Bassel-Duby R, Olson EN (2011). Mice lacking microRNA 133a develop dynamin 2-dependent centronuclear myopathy. J Clin Invest.

[47] Guo X, Guo L, Ji J, Zhang J, Zhang J, Chen X, Cai Q, Li J, Gu Q, Liu B, Zhu Z, Yu Y (2010). miRNA-331-3p directly targets E2F1 and induces growth arrest in human gastric cancer. Biochem Biophys Res Commun.

[48] van’t Hof FN, Ruigrok YM, Baas AF, Kiemeney LA, Vermeulen SH, Uitterlinden AG, Hofman A, Rivadeneira F, Rinkel GJ, de Bakker PI (2013). Impact of inherited genetic variants associated with lipid profile, hypertension, and coronary artery disease on the risk of intracranial and abdominal aortic aneurysms. Circ Cardiovasc Genet.

[49] Scheper GC, van der Knaap MS, Proud CG (2007). Translation matters: protein synthesis defects in inherited disease. Nat Rev Genet.

[50] Casad ME, Abraham D, Kim IM, Frangakis S, Dong B, Lin N, Wolf MJ, Rockman HA (2011). Cardiomyopathy is associated with ribosomal protein gene haplo-insufficiency in Drosophila melanogaster. Genetics.

[51] Hannan RD, Stefanovsky V, Taylor L, Moss T, Rothblum LI (1996). Overexpression of the transcription factor UBF1 is sufficient to increase ribosomal DNA transcription in neonatal cardiomyocytes: implications for cardiac hypertrophy. Proc Natl Acad Sci U S A.

[52] Mourani PM, Garl PJ, Wenzlau JM, Carpenter TC, Stenmark KR, Weiser-Evans MC (2004). Unique, highly proliferative growth phenotype expressed by embryonic and neointimal smooth muscle cells is driven by constitutive Akt, mTOR, and p70S6K signaling and is actively repressed by PTEN. Circulation.

[53] Hegner B, Lange M, Kusch A, Essin K, Sezer O, Schulze-Lohoff E, Luft FC, Gollasch M, Dragun D (2009). mTOR regulates vascular smooth muscle cell differentiation from human bone marrow-derived mesenchymal progenitors. Arterioscler Thromb Vasc Biol.

[54] Ma XM, Blenis J (2009). Molecular mechanisms of mTOR-mediated translational control. Nat Rev Mol Cell Biol.

[55] Rodier F, Campisi J, Bhaumik D (2007). Two faces of p53: aging and tumor suppression. Nucleic Acids Res.

[56] Kim R (2005). Unknotting the roles of Bcl-2 and Bcl-xL in cell death. Biochem Biophys Res Commun.

[57] Hara A, Yoshimi N, Mori H (1998). Evidence for apoptosis in human intracranial aneurysms. Neurol Res.

[58] Takagi Y, Ishikawa M, Nozaki K, Yoshimura S, Hashimoto N (2002). Increased expression of phosphorylated c-Jun amino-terminal kinase and phosphorylated c-Jun in human cerebral aneurysms: role of the c-Jun amino-terminal kinase/c-Jun pathway in apoptosis of vascular walls. Neurosurgery.

[59] Laaksamo E, Tulamo R, Baumann M, Dashti R, Hernesniemi J, Juvela S, Niemela M, Laakso A (2008). Involvement of mitogen-activated protein kinase signaling in growth and rupture of human intracranial aneurysms. Stroke.

[60] Weinsheimer S, Lenk GM, van der Voet M, Land S, Ronkainen A, Alafuzoff I, Kuivaniemi H, Tromp G (2007). Integration of expression profiles and genetic mapping data to identify candidate genes in intracranial aneurysm. Physiol Genomics.

[61] Frosen J, Piippo A, Paetau A, Kangasniemi M, Niemela M, Hernesniemi J, Jaaskelainen J (2006). Growth factor receptor expression and remodeling of saccular cerebral artery aneurysm walls: implications for biological therapy preventing rupture. Neurosurgery.

[62] Ruigrok YM, Baas AF, Medic J, Wijmenga C, Rinkel GJ (2012). The transforming growth factor-beta receptor genes and the risk of intracranial aneurysms. Int J Stroke.

[63] Peters DG, Kassam AB, Feingold E, Heidrich-O'Hare E, Yonas H, Ferrell RE, Brufsky A (2001). Molecular anatomy of an intracranial aneurysm: coordinated expression of genes involved in wound healing and tissue remodeling. Stroke.

